# State-of-the-Art Analytical Approaches for Illicit Drug Profiling in Forensic Investigations

**DOI:** 10.3390/molecules27196602

**Published:** 2022-10-05

**Authors:** Reem Ahmed, Mohamad J. Altamimi, Mayssa Hachem

**Affiliations:** 1 Department of Chemistry, Khalifa University, Abu Dhabi P.O. Box 127788, United Arab Emirates; 2 Dubai Police, General Department of Forensic Science and Criminology, Forensic Chemistry Section, Dubai P.O. Box 1493, United Arab Emirates; 3 Department of Chemistry and Healthcare Engineering Innovation Center, Khalifa University, Abu Dhabi P.O. Box 127788, United Arab Emirates

**Keywords:** illicit drugs, drug profiling, analytical approaches, forensic investigation

## Abstract

In forensic chemistry, when investigating seized illicit drugs, the profiling or chemical fingerprinting of drugs is considered fundamental. This involves the identification, quantitation and categorization of drug samples into groups, providing investigative leads such as a common or different origin of seized samples. Further goals of drug profiling include the elucidation of synthetic pathways, identification of adulterants and impurities, as well as identification of a drug’s geographic origin, specifically for plant-derived exhibits. The aim of this state-of-art-review is to present the traditional and advanced analytical approaches commonly followed by forensic chemists worldwide for illicit drug profiling. We discussed numerous methodologies for the physical and chemical profiling of organic and inorganic impurities found in illicit drug. Applications of powerful spectroscopic and chromatographic tools for illicit drug profiling including isotope-Ratio mass spectrometry (IRMS), gas chromatography–mass spectrometry (GC-MS), gas chromatography–isotope ratio mass spectrometry (GC-IRMS), ultra-high-performance liquid chromatography (UHPLC), thin layer chromatography (TLC), liquid chromatography–mass spectrometry (LC-MS) and inductively coupled plasma-mass spectrometry (ICP-MS) were discussed. Altogether, the techniques covered in this paper to profile seized illicit drugs could aid forensic chemists in selecting and applying a suitable method to extract valuable profiling data.

## 1. Introduction

Over the past ten years, illicit drug markets have been growing exceedingly worldwide. By 2015, illicit drugs accounted for the largest volume of criminal cases analyzed by forensic laboratories [1]. Even though extensive research has covered the possible detrimental health complications of using these drugs, illicit drugs remain a significant problem globally. According to the United Nations Office on Drugs and Crime (UNODC), drug consumption killed half a million of people in 2019, while drug use disorders killed 18 million healthy individuals. In 2020 alone, around 275 million people have consumed illicit drugs with a 10% increase compared to 2010. By 2030, this number is expected to increase by 11% worldwide [2].

Moreover, UNODC highlighted the lack of public awareness about the possible risks of consuming drugs in some countries. For instance, the number of teenagers who do not perceive marijuana as harmful has increased globally by 40% [2].

Generally, illicit drugs can originate from natural sources (cannabis and cocaine), be prepared from natural materials (in the case of heroine) or can be synthetic (amphetamine-type substances ATS) [3]. Illicit drugs are associated with significant health risks. These drugs can either inhibit or stimulate the central nervous system (CNS) and may have hallucinogenic effects. Table 1 shows a non-extensive list of several illicit drugs and their categories based on their effects on the CNS: stimulants, depressants, and hallucinogens. In addition to these effects, illicit drugs might cause physical and psychological dependence, especially with long-term use [4].

Recently, an increase in the number of illicit drug seizures worldwide was observed. For example, the quantity of seized cannabis resin (hashish) has shown an upward trend after a decrease in 2018 [5]. Coca bush cultivation, targeted for cocaine extraction, has also increased steadily from 2016 to 2019 [6].

In the United States, between 2012 and 2019, a significant increase in the number of people who died due to cocaine overdose was observed [6]. Moreover, amphetamine-type-Substance (ATS) seizures increased tremendously from 2009 to 2019 [6].

In addition to illicit drug market growth, another major challenge for law enforcement and forensic investigators is the encountering of new psychoactive substances (NPS) that may have not yet have been included in drug schedules and are frequently synthesized at high rates [2]. For instance, UNODC has reported that in South and Central America alone, the number of NPS seizures increased five-fold between 2015 and 2019 [2].

To address the mentioned challenges, forensic chemists should follow specific approaches and develop new methodologies in forensic chemistry for drug profiling.

Generally, profiling of a drug involves gathering all of its chemical and physical characteristics [7]. Indeed, physical profiling usually refers to a drug’s packaging and general appearance [8], whereas chemical profiling is more complex and requires a variety of analytical techniques to gather data about an illicit substance, other agents/excipients (e.g., adulterant and diluents), by-products, precursors, solvents, and impurities [4].

In forensic investigations, drug profiling provides information about a drug’s origin and links between seizures. More recently, drug profiling facilitated the development of sensors and fast screening methods used by police agencies.

In this state-of-the-art review, first, physical profiling of illicit drugs through gathering of all possible physical characteristics of a particular seized drug is discussed.

In forensic investigations of seized illicit drugs, physical profiling alone does not provide sufficient data. Hence, the importance of introducing chemical profiling, including organic and inorganic profiling, is clear.

For organic profiling of illicit drugs, several analytical approaches are followed. These approaches include isotope-ratio mass spectrometry (IRMS), gas chromatography–mass spectrometry (GC-MS), gas chromatography–isotope ratio mass spectrometry (GC-IRMS), ultra-high-performance liquid chromatography (UHPLC), thin layer chromatography (TLC) and liquid chromatography–mass spectrometry (LC-MS). The main approach used for the inorganic or elemental profiling of illicit drugs is via inductively coupled plasma–mass spectrometry (ICP-MS) analysis. Altogether, the mentioned approaches cover all the techniques used to profile organic and inorganic impurities when investigating seized illicit drugs.

## 2. Physical Profiling of Illicit Drugs

Physical profiling of illicit drugs refers to gathering all possible physical characteristics about a particular seized drug. This includes the color, packaging material, thickness of packaging plastic, logo on either tablets or package as well as tablets’ weight and dimensions [9].

Physical drug profiling provides complementary information that may later support the chemical profiling of a drug. It may allow for the grouping of illicit drugs as well as speculation as to whether certain groups originate from a similar source or not. For instance, if a batch of 3,4-methylenedioxymethamphetamine (MDMA) tablets or heroin blocks were pressed with a tool with some imperfection, then that imperfection would be printed on the whole batch. Examination of this detail might provide evidence that these drugs probably have the same source [10].

Chank-W et al., 2012 examined in-depth more than 300 heroin samples focusing on five different physical characteristics including the color and weight of the substance and the width, weight, and thickness of plastic package. Researchers showed that film thickness was the least-reliable characteristic due to huge variability between samples. On the other hand, the dimensions of the package were the most reliable characteristic, and this was suggested as a trademark for a certain production line. By excluding film thickness, they suggested a similarity between all samples concluding that they may originate from the same source. Besides physical profiling, researchers examined the chemical profiles of heroin samples for further confirmation and suggested that samples may share, at least, two common sources [11].

Additionally, the physical characteristics of MDMA tablets were investigated. Links were established between samples, even those with different external characteristics. Researchers suggested that differences in seizures’ times across countries might cause non-production-related differences or similarities between samples. This is one of the main drawbacks of relying on physical information alone where more information is needed to better understand the trafficking process [9].

**Figure 1:**
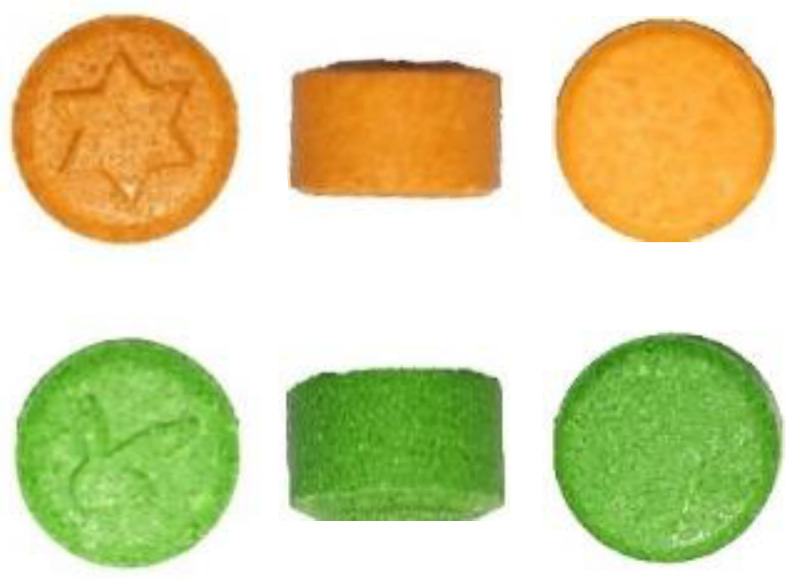
MDMA tablets from street samples provided by Forensic Chemistry Section, General Department of Forensic Science and Criminology, at Dubai Police. Samples from the same seizure revealed physical similarities (diameter of 8.22 mm, measured using a calibrated Mitutoyo Caliper). However, tablets exhibited different logos and colors.

As shown in Figure 1, MDMA tablets from street samples, the same seizure analyzed at Forensic Chemistry Section, General Department of Forensic Science and Criminology, at Dubai Police, Dubai, United Arab Emirates, exhibited similar physical characteristics with an 8.22 mm diameter but different logos and colors (unpublished data).

Other researchers found that a group of physically dissimilar ecstasy (MDMA) tablets have very similar chemical characteristics, suggesting that they may share a common source [12].

Indeed, the physical profiling revealing no similarity may be due to manufacturers using diverse concealment approaches to eliminate any physical evidence that could link samples [10]. Additionally, these dissimilarities may be due to uncontrolled clandestine laboratory conditions producing variation in drug’s physical characteristics [13]. Thus, utilizing chemical profiling techniques becomes necessary.

## 3. Chemical Profiling of Illicit Drugs

Chemical profiling of a drug consists of gathering all chemical information about drug. It can be classified into organic and inorganic profiling based on the analytical technique applied for drugs’ analysis and type of impurity.

Table 2 and Table 3 respectively summarize common approaches widely accepted in the forensic community and court with references followed for organic and inorganic profiling of drugs.

For organic profiling, several analytical approaches were beneficial for analyzing organic impurities. These impurities might originate from different sources including the natural source of the drug, its environment, the process of drug synthesis, the drug’s packaging, as well as any cutting agents. Cutting agents are added to the active ingredient in order to increase the weight of the sold substance and adulterants are added to enhance or add psychoactive effects to the substance [13].

For forensic investigation purpose, some countries own programs that specify chemical fingerprints or signatures. These signatures are specified for common illicit drugs and can suggest appropriate techniques for their analysis.

For example, in Australia, specific signatures for amphetamine-type substances (ATS), heroin, and cocaine are established [10]. ATS have two main signatures (I, II). For signature I, forensic examiners analyze seized samples for their by-product content. This examination enables an understanding of the synthetic route and precursors using GC-MS [10]. For signature II, investigators analyze elemental traces, originating from catalysts, using ICP-MS [10]. These traces might reveal information about illicit drugs’ synthetic routes [10]. Other countries may have similar criteria or slightly modified ones.

### 3.1. Organic Chemical Profiling

#### 3.1.1. Isotope-Ratio Mass Spectrometry

Isotope-ratio Mass Spectrometry (IRMS) is considered a powerful tool in forensic investigations regarding drug profiling. Many methodologies are based on the hypothesis that natural illicit drugs, mainly those collected from plants, exhibit an IRMS profile reflecting the plants’ environmental and growth conditions. This may help provide information about the origin of the illicit drug.

In 2006, researchers identified links between provinces in Brazil through seized samples of marijuana based on the analysis of carbon and nitrogen isotopes related mostly to climate and other environmental plant growth conditions [14]. In addition, the nitrogen isotope was used to analyze big cocaine seizures in 2007 when researchers linked certain logos to a group of samples but could not establish any correlations [15]. However, they were able to find significant variations in nitrogen isotope and linked most negative δ^15^NAIR to successive precipitation of cocaine-HCl [15]. Through comparison, researchers recognized geographical links between samples of known origin and a characteristic range of δ^15^NAIR for samples from a particular location [15].

In 2009, other researchers could identify whether marijuana was grown in indoor or outdoor conditions by focusing on carbon and nitrogen isotopes [16]. Indeed, nitrogen isotopes provide indications about the fertilizers used during the growth process of marijuana plants. Although scientists were not able to identify strong geographic links, they suggested using a control source sample to compare and establish geographical evidence [16].

In addition to drugs’ growth environment evidence, IRMS analysis can reveal information related to residue intermediates and precursors, commonly found and collected from clandestine laboratories [17]. More recently, IRMS profiled MDMA and 3,4-Methylenedioxyamphetamine (MDA) precursors, previously reported in clandestine laboratories [18]. Significant differences in isotopic profiles were observed, leading to grouping and discriminating precursors, as well as MDMA/MDA synthesized from them [18].

Additionally, MDMA samples were analyzed via IRMS with the aim of grouping drug samples based on their synthetic route. Scientists found that hydrogen isotope was the best way to provide three different groups based on three different synthetic routes [19]. However, carbon and nitrogen were not useful for grouping samples based on synthesis. Nitrogen could be affected by reaction mechanisms and group samples based on production batches rather than their synthetic route [19]. Researchers suggested studying the effects of pressure, temperature, and stirring time on the IRMS profile of the drugs [19].

Moreover, benzylpiperazine hydrochloride (BZP. HCl), an emerging drug of abuse, was analyzed using IRMS through δ^13^C and δ^15^N isotopic data analysis [17]. Researchers reported that when samples had different suppliers, isotopic profiles could differentiate between samples and/or intermediates [17].

**Figure 2:**
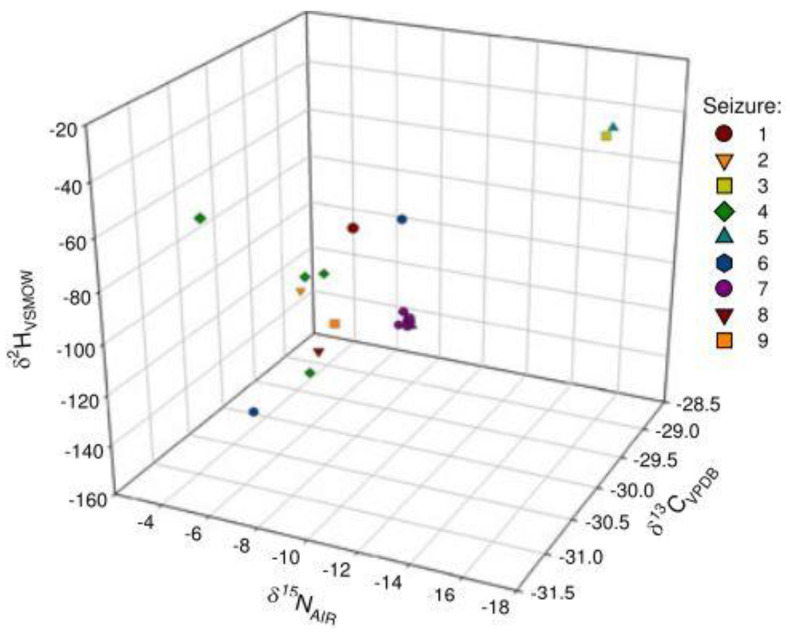
Multivariate representation of δ^13^C, δ1^5^N, and δ^2^H values of target seized cathinones between 2009 and 2014 [20]. “Reprinted with permission from Ref. [20]. Copyright 2022, John Wiley and Sons”. Copyright Clearance Center License Number 5366311075145.

Since IRMS has shown its ability to profile drugs and reveal links, recent research focuses on applying IRMS for modern challenges. For example, researchers analyzed a cathinone, a subgroup of NPS, using isotopic ratios of δ^13^C, δ^15^N, and δ^2^H and linked samples with similar synthetic origin [20]. As illustrated in Figure 2, Seizures 7, 8 and Seizures 3, 5 have identical isotopic ratios indicating that these seizures may have a similar synthetic origin [20].

Furthermore, researchers analyzed cannabinoid 5F-PB-22, a synthetic cannabinoid and another class of NPS, using IRMS. Two extraction methods were applied to the cannabinoid from herbal samples without significantly affecting the drug’s profile [21]. Isotopic ratio measurements were helpful in linking different 5F-PB-22 batches and assigning pure materials to herbal blends [21].

#### 3.1.2. Application of GC-MS

GC-MS is the gold standard for illicit drug profiling. Several GC-MS methodologies have been developed to support forensic investigations.

GC-MS has been incorporated in the Australian Illicit Drug Intelligence Program since 2007 to identify two heroin signatures, two cocaine signatures, and two signatures for amphetamine-type substances [10].

Furthermore, in 2015, researchers in Finland used GC-MS to chemically profile cocaine samples. They identified the diluents, cocaine purity, and chemical profile of the seized cocaine samples in a single injection. Their methodology allowed for differentiation between samples originating from different sources. These results identified trafficking routes as well as distribution networks [22].

In addition, GC-MS has been utilized to profile over 500 seized heroin samples from different origins. Samples were classified into subgroups depending on the analysis of neutral and acidic impurities. Researchers suggested that examining trace levels of these impurities could be linked to geographical origins and might allow for the development of the heroin signature program [23].

More recently, large cocaine seizures from a common source were studied using GC-MS. Researchers examined residual solvents’ profiles as well as alkaloids’ profiles. The alkaloid profiles revealed significant differences, while the residual solvents profile presented little variation in profiles. Thus, researchers suggested that although differences in the alkaloid profiles were more significant, the residual solvent profiles could still link samples to a common provenance [24]. Other scientists have also reported that GC-MS was well suited as a single technique to detect links between seizures and could be used as a standard method for operational intelligence purposes [25].

Looking to recent international research, researchers are hurtling toward developing new methodologies to use GC-MS for drug profiling and assisting forensic investigations.

**Figure 3:**
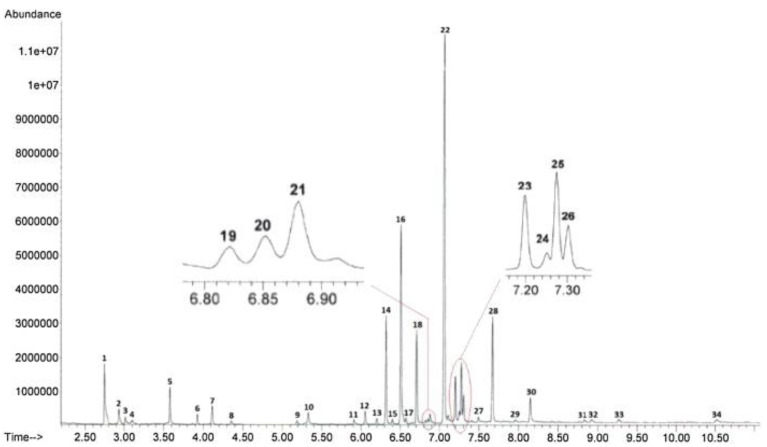
Typical chromatograms of the actual street nyaope samples analyzed through GC-MS. (1) nicotine; (2) α-caryophyllene; (3) α-humelene; (4) α/β-selinene; (5) phenacetin; (6) neophyltadiene; (7) caffeine; (8) palmitic acid; (9) phytol; (10) (Z,Z,Z)-9,12,15-octadecatrienoic acid; (11) cannabivarol; (12) 4,8,13-duvatriene-1,3-diol; (13) cannabicyclol; (14) tetrahydrocannabivarin; (15) cannabichromene; (16) internal standard tetracosane; (17) cannabivarin; (18) cannabidiol; (19) nevirapine; (20) cannabicoumaronone; (21) unknown; (22) Δ9-tetrahydrocannabinol; (23) cannabigerol; (24) acetylcodeine; (25) cannabinol; (26) 6-monoacetylmorphine; (27) heneicosane; (28) diamorphine; (29) squalene; (30) 11-butyl docosane; (31) unknown; (32) triacontane; (33) vitamin E and (34) β/γ-sitosterol [26]. “Reprinted with permission from Ref. [26]. Copyright 2022, Elsevier”. Copyright Clearance Center License Number 5386920362795.

**Figure 4:**
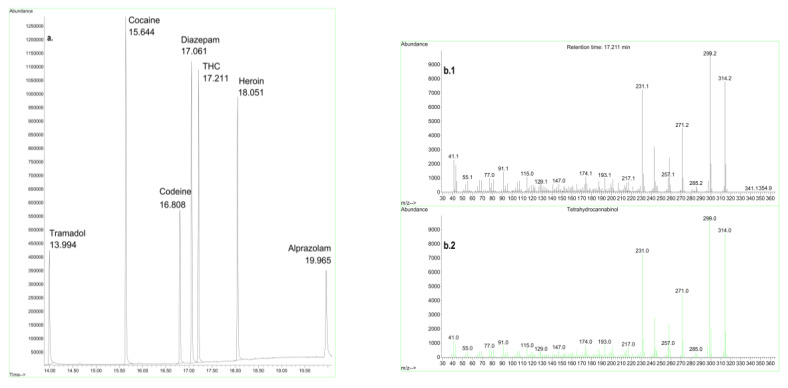
GC-MS chromatogram of multiple drugs of abuse (**a**), in addition to the mass spectrum of THC (**b1**) (tetrahydrocannabinol, which is the psychoactive ingredient in marijuana) and a spectral library match for THC (**b2**).

Figure 3 and Figure 4 represent, respectively, illustrations of typical chromatographs for street nyaope samples analyzed by GC-MS and a simultaneous GC-MS analysis of multiple drugs of abuse.

For instance, GC-MS characterized nyaope, a common drug in South Africa [26]. This drug consists mainly of low-grade heroin, antiretroviral drugs, cannabis products, and some diluents. GC-MS is appropriate for the analysis of nayope samples which comprise acidic and basic drugs volatile under GC-MS environments without the necessity for samples’ derivatization. Chromatographic separation was achieved with a fused-silica capillary column HP-5MS (30 m × 0.25 mm, film thickness 0.25 mm). Splitless injection was used at 280 °C. High-purity helium was used as the carrier gas, at a flow rate of 1 mL/min. MS analysis was performed with electron ionization (EI) mode at 70 eV and mass spectrometer (quadrupole) in scan mode. Samples’ analysis was completed in the presence of the internal standard tetracosane [26]. The abundancy of peaks in Figure 3 indicates ratios of different components in the sample. Through reference standards, quantification is also possible. Each peak represents a component of the sample eluting from the GC column. This component is detected by a mass spectrometer that provide a unique fragmentation pattern to elucidate the structure of the eluent. Researchers concluded that tertiary butyl alcohol is the best solvent to use for the identification, comparison, and profiling of nyaope samples [26].

Figure 4 is another example where the GC-MS approach could separate and identify multiple components in a seized sample of drugs of abuse (unpublished data provided by Forensic Chemistry Section, General Department of Forensic Science and Criminology, at Dubai Police, Dubai, United Arab Emirates). Figure 4a illustrates a GC chromatogram of multiple drugs of abuse in a prepared standard sample (tramadol, cocaine, codeine, diazepam, THC, heroin, and alprazolam). In the sample, a peak at 17.211 min was observed. To identify the substance eluted at 17.211 min, MS was applied (Figure 4(b1)). The fragmentation patterns of the questioned sample (Figure 4(b1)) were compared to those of standard THC previously analyzed and saved in the reference library of drugs of abuse at Dubai Police (Figure 4(b2)). The similarities between the fragmentation patterns of the questioned and standard sample confirmed that the eluting component at 17.211 min was THC. These illustrations confirm why forensic laboratories consider GC-MS the gold standard technique in the analysis of drugs of abuse.

In 2019, GC-MS was modified with time-of-flight–mass spectrometry (TOF-MS) instead of traditional MS and used for NPSs screening. TOF-MS screened more than 50 NPS samples in 10 min. Sixty-three seized samples were screened in order to develop this method. Researchers determined that “The method was able to separate and identify new-generation NPS that were not included in the list of standards used to develop the method” [27].

In 2021, GC-MS was chosen to profile a huge quantity of street drug seizures (5647 samples). This study provided helpful guidelines for the development of portable sensors [28]. Chemical or analytical fingerprints could be sensed to detect certain illicit compounds like Δ-9-tetrahydrocannabinol (THC), a substance found in marijuana, hashish, and hashish oil. They also reported some interfering substances that may affect the sensing process of substances (e.g., other alkaloids).

##### GC-IRMS

GC-IRMS is a combination of both gas chromatography and IRMS approaches where the sample passes through a GC instrument and is then directed into IRMS where it is further separated and identified by a detector based on m/z values. In general, IRMS is preferable in comparison to GC-IRMS due to the ease of handling, less potential for malfunction, and speed [29].

However, when the amount of sample is less than 6–8 mg (minimum amount needed for IRMS analysis), GC-IRMS is suggested to be more appropriate [29].

Some research was conducted on the application of GC-IRMS for illicit drug profiling. Scientists have applied GC-IRMS to examine hemp leaves, heroin, morphine, and cocaine and suggested that GC-IRMS is suitable for large samples with impurities [30].

Additionally, the carbon isotope ratios of heroin samples could be analyzed using GC-IRMS [31]. Researchers found that carbon isotope ratios vary depending on geographical location of production [31]. Another study compared elemental analysis—isotope ratio mass spectrometry (EA-IRMS) and GC-IRMS for the analysis of synthetic cannabinoids [29]. Despite the higher uncertainty associated with GC-IRMS measurements, both instrumentations were comparable and suitable for drug profiling [29].

#### 3.1.3. UHPLC/HPLC and TLC

**Figure 5:**
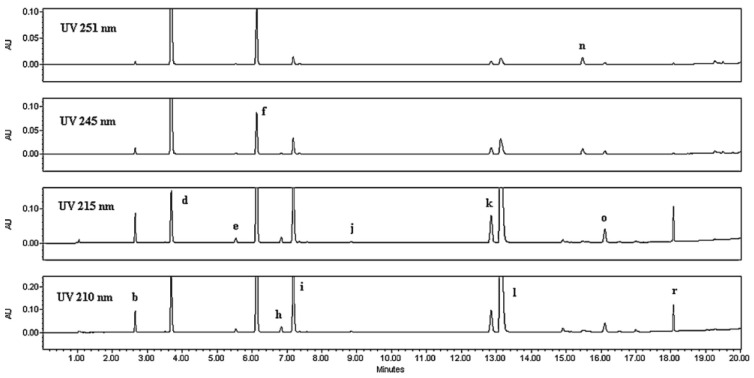
UHPLC separation of a sample categorized as southwest Asian heroin. UHPLC separation of a standard mixture of: (b) morphine, (d) acetaminophen, (e) codeine, (f) caffeine, (h) O3-monoacetylmorphine, (i) O6-monoacetylmorphine, (j) lidocaine, (k) acetylcodeine, (l) heroin, (n) papaverine, (o) noscapine, and (r) methorphan [32]. “Reprinted with permission from Ref. [32]. Copyright 2022, Elsevier”. Copyright Clearance Center License Number 5387441285219.

Ultra-high-performance liquid chromatography (UHPLC) can organically profile illicit drugs. The technique was reported to be suitable for heroin profiling [32]. Through UHPLC analysis, impurities as low as 0.02%, with respect to heroin, were detected and used for origin identification [32]. The determination of heroin classified to be from Southwest Asia (SWA) and seven frequently encountered impurities (morphine, O3-monoacetylmorphine, O6-monoacetylmorphine, codeine, acetyl codeine, noscapine, and papaverine) using reversed-phase UHPLC with PDA UV detection was described [32]. For the quantification of heroin and impurities, separations were conducted with a C18 or CSH Fluoro-Phenyl 1.7 mm particle column (150 mm × 2.1 mm). This method showed an overall agreement for classification purposes with a certified capillary electrophoresis method. Such impurities were supportive in classifying samples based on geographical location [32]. Figure 5 illustrates a multi-wavelength UHPLC chromatogram of a sample classified as SWA and contaminated with acetaminophen, caffeine, lidocaine, and methorphan [32].

HPLC along with GC-MS supported in the assessment of illicit drugs’ quality [33]. Researchers used this approach to examine cutting agents in samples of heroin and cocaine [33].

Another liquid chromatography technique valuable for the analysis of illicit drugs is thin layer chromatography (TLC). TLC is usually used to recognize different components of illicit drugs. However, in order to provide complete and adequate drug profiling, TLC is usually combined with other techniques. Heroin samples were analyzed using different techniques including TLC to identify various components of samples [34]. Using TLC, caffeine was detected in heroin samples, suggesting that heroin samples are most likely from Southwest Asia [34].

#### 3.1.4. Liquid Chromatography–Mass Spectrometry (LC-MS)

**Figure 6:**
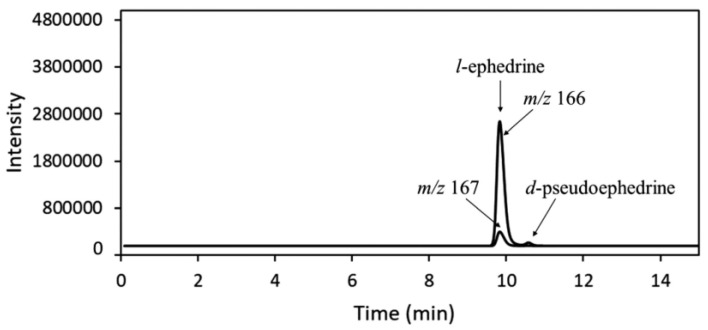
SIM chromatogram of l-ephedrine/HCl [35]. “Reprinted with permission from Ref. [35]. Copyright 2022, John Wiley and Sons”. Copyright Clearance Center License Number 5387451131729.

For illicit drug investigations, LC-MS has been employed to identify whether it can replace IRMS since it is more routinely available in laboratories. Through LC-MS analysis, information on the origin of ephedrine and pseudoephedrine, precursors of MDMA, could be determined [35]. Based on the method by means of authenticated standard samples in IRMS, a standard sample for obtaining a stable isotopic ratio by LC/MS was designated. The abundance ratio of the [M + 2H]^+^ ion to the [M + H]^+^ ion was determined through selected ion monitoring (SIM). Figure 6 illustrates an example of SIM chromatogram for one of the analyzed samples (l-ephedrine/HCl). For LC conditions, Poroshell 120-ECC18 column (2.1mm × 150 mm, 2.7 μm) and eluent isocratic mode with a flow rate of 0.3 mL/min, 6% acetonitrile/94% 20 mM ammonium formate solution were used. Electrospray ionization in positive ion mode was followed for MS conditions.

A value (δR_sample_) was defined to compare the abundance ratio of stable isotope ion [M + 2H]^+^ to protonated ion [M + H]^+^ in the analyzed samples. This value was calculated by implementing the height and/or the area under SIM chromatograph [35]. Final δR values were compared to stable isotope ratios (δ^2^H, δ^13^C, and δ^15^N) to establish correlations [35]. Through LC-MS analysis, the stable isotope ratio obtained for ephedrines had good correlation with carbon and hydrogen stable isotope ratios identified through IRMS [35].

Furthermore, new LC-MS methods were developed for NPS profiling such as the electrochemical profiling technique [36]. This method allows for categorization based on oxidation mechanism. The approach was reported as successful and may be helpful to police agencies [36].

### 3.2. Inorganic Chemical Profiling through Inductively Coupled Plasma–Mass Spectrometry (ICP-MS)

The ICP-MS technique has many applications in forensic chemistry including the elemental profiling of soil samples, relating soil samples to different locations, determining the origins and production sites of different glass samples, and elemental analysis of different types of evidence collected from a crime scene, such as inks, dyes, pigments, additives, and stabilizers [37,38].

In illicit drug profiling, ICP-MS is considered to be a very powerful approach, since it offers low detection limits and covers any element in the periodic table [39]. Metal traces might exist in drug tablets due to the origin of plants used in producing the drug, the production method, the production containers, as well as air-borne particles [40].

ICP-MS was used for the analysis of a myriad of illicit drugs including heroin, 3,4-Methylenedioxymethamphetamine (MDMA), and cocaine. Researchers suggested ICP-MS for the analysis of heroin samples originating from the golden crescent (Afghanistan) and golden triangle (Myanmar) [41]. They analyzed 19 different elements, and 10 elements were found to significantly differ between samples from the golden crescent and golden triangle [41]. Subsequently, they also investigated 907 heroin samples with unknown origins [41]. For comparison, two organic profiling methods (CE-DAD and GC-MS) and the ICP-MS technique were applied, and good correlation was determined [41]. They concluded that the ICP-MS method was faster compared to organic profiling techniques, more environmentally friendly, and more beneficial when screening large quantities [41]. Moreover, other researchers determined the provenance of 88% of the total heroin samples analyzed. This was done through ICP-MS analysis of several elements including Na, Mg, Cr, Fe, Zn, Zr, Cd, Pb, and U [42]. Significant differences for these elements were observed between heroin originating from East Asia and Eastern Hemisphere America as well as differences between Southeast Asia vs. Southwest Asia and Mexico vs. South America [42].

ICP-MS has been used to examine cocaine seizures from three different areas in a small state in Brazil. Both ICP-MS and inductively coupled plasma–optical emission spectrometry (ICP-OES) were used to detect and quantify diverse elements in cocaine samples, mainly Al, Ca, Cu, Fe, Mn, Mg, Zn, Mo, Co, Pb, and PP [43]. Cocaine samples had homogenous inorganic profiles and similar concentrations, mainly due to geographic proximity [43].

ICP-MS was successfully used in the inorganic profiling of 37 cocaine samples, and examined 10 elements including Pb, Cu, Mg, Mn, Cr, As, Ni, Fe, Co, and Ca [44].

Moreover, ICP-MS analysis of 183 cocaine samples for 26 different elements was followed by mathematical analysis to investigate potential links between cocaine seizures [45]. Results showed that cocaine samples were distributed among 21 different groups and links between and within provinces in China were established [45].

In forensic investigations, another illicit drug analyzed through ICP-MS is MDMA. Researchers investigated 150 MDMA samples (ecstasy tablets) seized from nine different cities in a state in Brazil [46]. Twenty-five different elements (including As, Ba, Bi, Ca, Cd, Ce, Co, Cu, Cs, Er, La, Mg, Mn, Mo, Nd, Ni, Pb, Pr, Rb, Se, Sb, Te, Tl, U, and Zn) were examined using ICP-MS [46]. By performing statistical analysis, MDMA samples were distributed between two main clusters, suggesting two main sources of MDMA tablets [46]. Nd, Ni, and Pb concentration values alone could predict the same result with 100% accuracy [46].

A similar study was conducted on seizures from two cities in the same state in Brazil with the aim being to discriminate between seizures originating from both. Researchers analyzed 25 elements using ICP-MS and found significant differences in certain elements among the 25 [47]. To predict the samples’ origin, a statistical classification model was created based on three elements: Se, Mo, and Mg. Interestingly, these three elements could differentiate between samples originating from two cities and provided 81.58% accuracy, 95.24% sensitivity, and 64.71% specificity to the model presented [47].

Other researchers analyzed MDMA using ICP-MS and inductively coupled plasma atomic emission spectroscopy (ICP-AES) with the aim of determining the synthesis method (reductive amination) based on catalysis. Traces of Pt and B were found in 88 out of 97 samples [48]. Results indicated that Pt and sodium borohydride (NaBH_4_) were used as catalysts which allowed for identification of the synthetic route [48]. None of samples were synthesized using the aluminum amalgam method where usually aluminum foil and mercury chloride (HgCl_2_) are used, and Hg particles are expected to show in the elemental profile [48]. Thirteen links were also determined among ninety-seven total seized MDMA samples [48].

NicDaéid et al. aimed to synthesize methamphetamine chloride (a drug of the amphetamines group with a similar effect to MDMA), through two different synthesis routes (hypophosphorous and Moscow) in order to mimic clandestine laboratories [49]. All the chemicals needed were from either household items or pharmaceutical medications available in UK [49]. Following this, the samples were analyzed through ICP-MS. Results suggested that Na and S concentrations could identify the salting-out process. Differences of P and I discriminated the route of synthesis, since the hypophosphorous method exhibited a higher elemental composition of both iodine and phosphorous [49].

Likewise, Bora and Çelikkan have studied MDMA samples with a full profile (physical and chemical). ICP-MS inorganic analysis involved seventeen elements including Li, Be, V, Cr, Co, Ni, Cu, Zn, As, Se, Rb, Sr, Ag, Cd, Cs, Ba, and Pb [12]. Ba provided valuable information about seizures, particularly when the EMDE route—a synthetic route to synthesize MDMA—was followed [12]. Based on statistical analysis, researchers suggested using Cu, Co, Rb, and Pb in addition to Ba for profiling seizures [12]. This cluster of elements might originate from a common source and aid in identifying a common source of seized samples [12].

Cannabis samples could also be analyzed by ICP-MS, distinguished based on the type of liquid nutrients used to grow the cannabis plant [50]. B, Cu, Mn, Rb, Sr, and Zn elemental composition were analyzed in cannabis samples and could be correlated to different commercial nutrients [50].

**Table 2:** Organic profiling of illicit drugs.

Organic Profiling	References
Isotope Ratio Mass Spectrometry	[14] Benson et al., 2006 [15] Sewenig et al., 2007 [16] West et al., 2009 [17] Beckett et al., 2015 [18] Cormick et al., 2021 [19] Hilary et al., 2008 [20] Collins et al., 2016 [21] Münster-Müller et al.,2018 [51] Brand Willi et al., 2014 [52] Gentile et al., 2015
Gas Chromatography-Mass Spectrometry	[10] Collins et al., 2007 [22] Broseus et al., 2015 [23] Morello et al., 2010 [24] Nielsen et al., 2017 [25] Morelato et al., 2014 [26] Mthembi et al., 2018 [27] Dei Cas et al., 2019 [28] Zubrycka et al., 2022
Gas Chromatography-Isotope Ratio Mass Spectrometry	[29] Münster-Müller et al., 2020 [30] Galimov et al., 2005 [31] Desage et al., 1991
Ultra-High-Performance Liquid Chromatography	[32] Lurie et al., 2013
High Performance Liquid Chromatography	[33] Bourmaud et al., 2021
Thin Layer Chromatography	[34] El-Neketi et al., 2018
Liquid Chromatography-Mass Spectrometry	[35] Makino et al., 2019 [36] Schram et al., 2021

**Table 3:** Inorganic profiling through Inductively Coupled Plasma–Mass Spectrometry of illicit drugs.

Inorganic Profiling	References
Inductively Coupled Plasma–Mass Spectrometry	[12] Bora et al., 2018 [37] Reidy et al., 2013 [38] Orellana et al., 2013 [39] Trueman et al., 2005 [40] Daéid et al., 2005 [41] Liu et al., 2014 [42] DeBord et al., 2018 [43] Amorim et al., 2021 [44] Bentil et al., 2019 [45] Liu et al., 2017 [46] Maione et al., 2017 [47] Maione et al., 2016 [48] Koper et al., 2007 [49] NicDaéid et al., 2013 [50] El-Deftar et al., 2015 [51] Brand Willi et al., 2014

## 4. Discussion and Conclusions

The profiling of illicit drugs is based on collecting all the potential physical and chemical characteristics of a specific drug allowing forensic laboratories to provide valuable forensic intelligence to police agencies.

For physical profiling, forensic chemists gather all of a drug’s physical characteristics without involving any chemical analysis. Generally, in forensic chemistry, these physical characteristics are useful for establishing a hypothesis but are not considered conclusive or acceptable on their own. On the other hand, the chemical profiling of illicit drugs is more powerful, since it employs numerous analytical approaches previously discussed.

Given the level of novel psychoactive substances (NPSs) on the market, through isotopic measurements, IRMS has been developed as a prospective procedure to assume the crop growing location of drugs, estimate trafficking means, and understand the synthetic pathways and chemicals used in clandestine laboratories. IRMS, in combination with developed chromatographic techniques such as GC, has provided developments in various areas of forensic science, and forensic chemistry is one of them. For trace amounts of sample, GC-IRMS is more appropriate than IRMS. One of the principal challenges in IRMS is the choice and use of suitable matrix harmonized and well-described reference materials (RMs) [52]. The last few years have seen important advances in RMs for the calibration of δ^2^H, δ^18^O, δ^13^C, δ^15^N, and δ^34^S values [51].

The gold standard for the chemical profiling of seized illicit drugs is GC-MS [52]. Numerous GC-MS approaches have been developed to reinforce forensic investigations as previously explained. Trace levels of drugs originating from different sources can be identified and links between seizures can be established.

Additionally, chromatographic techniques such as HPLC/UHPLC, TLC, and LC-MS are efficient in organically profiling traces of illicit drugs. However, LC-MS or HPLC could be the methods of choice when the sample to be analyzed is not enough volatile.

To investigate the inorganic or elemental profile of seized drugs, ICP-MS has been shown to be a powerful tool commonly used in forensic chemistry because it allows for the detection of trace levels of elements from the periodic table at parts-per-billion (ppb) level. The technique seems to provide information that is more valuable when the drug is synthetic. Contrarily, for natural illicit drugs, analyzing organic impurities may be more effective. An alternative technique called neutron activation analysis (NAA) is newly emerging in the field of elemental profiling. One research study reported both qualitative and quantitative data on 23 different elements in drugs and was able to differentiate between legal and illegal drug products [53].

Many of the analytical techniques used for drug analysis mentioned in this paper cannot be used at the scene where the sample to be investigated originated. Hence, there is a need for developing portable devices that can provide useful data in the field. Forensic applications for portable GC systems are relatively new for drug analysis, their major limitations are the electrical requirements and the carrier gases employed.

There are several other spectroscopic techniques for the analysis of illicit drugs including ultraviolet-visible (UV-Vis), infrared (IR), Raman and scanning electron microscope coupled with energy-dispersive X-ray (SEM-EDX) spectroscopy that are well known in the literature. However, to be accepted in court, these techniques must be broadly acknowledged as appropriate for the particular type of drug in question. It must be noted that the development of suitable spectral databases remains a critical issue for the effective use of these spectroscopic techniques. Spectral libraries continue to expand and must accommodate new forms of materials likely to be encountered. For example, the continuing evolution of illicit synthetic drugs means that the spectra of new chemicals must be included in databases to ensure effective identification.

The myriad of techniques available can also pose another issue, as data may not be comparable if different methodologies are used. This can be the case with heroin and cocaine profiling for example, where samples are profiled differently depending on the region in which they are being analyzed [54].

To help law enforcement and forensic investigators face all the challenges related to illicit drug trafficking and production, scientists should continuously develop new approaches to tackle the emerging types of illicit drugs. Finally, the authors suggest the emergence of an international collaborative initiative of forensic scientists to set standard profiling methodologies when investigating illicit drugs. This initiative could greatly enhance profiling efforts globally, especially since drug networks can span over a large area thousands of kilometers from the manufacturer’s location.

## Figures and Tables

**Table 1:** Classification of common illicit drugs based on their mechanism of action on the CNS.

Impact on CNS	Drug	Chemical Structure
**Stimulants**	Amphetamine	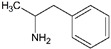
	Methamphetamine	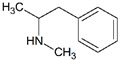
	3,4-methylenedioxyamphetamine (MDA)	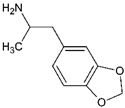
	3,4-methylenedioxymethamphetamine (MDMA)	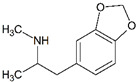
	Cocaine	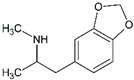
**Depressants**	Barbiturates	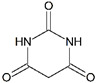
	Benzodiazepines	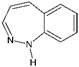
	Heroin	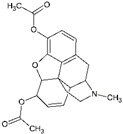
**Hallucinogens**	Ecstasy (3,4-methylenedioxymethamphetamine (MDMA))	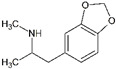
	Lysergic acid diethylamide (LSD)	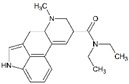
	Cannabis (Δ^9^-THC)	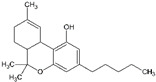

## Data Availability

Not applicable.
